# The negative association between inflammatory bowel disease and Helicobacter pylori seropositivity

**DOI:** 10.22088/cjim.10.2.217

**Published:** 2019

**Authors:** Rasoul Sayar, Javad Shokri Shirvani, Karimollah Hajian –Tilaki, Zeinab Vosough, Mohammad Ranaei

**Affiliations:** 1 Student Research Committee, Babol University of Medical Sciences, Babol, Iran; 2 Cancer Research Center, Health Research Institute, Babol University of Medical Sciences, Babol, Iran; 3 Social Determinants of Health Research Center, Health Research Institute, Babol University of Medical Sciences, Babol, Iran; 4 Clinical Research Development Center, Ayatollah Rouhani Hospital, Babol University of Medical Sciences, Babol, Iran

**Keywords:** Inflammatory Bowel disease, Helicobacter pylori, Enzyme-Linked immunosorbent assay, Immunoglobulin A, Immunoglobulin G

## Abstract

**Background:**

The role of Helicobacter pylori (H. pylori) in inflammatory bowel disease is a controversial argument. The initial theory of this study was that Helicobacter is a risk factor for inflammatory bowel disease. In this study, we investigated the coincidence of H. pylori exposure and IBDs.

**Methods:**

This case-control study has been done in Babol, teaching Hospitals; 60 newly diagnosed IBD cases without any Helicobacter eradicating treatment and 120 control patients without inflammatory bowel disease evidence in biopsy, investigated for H. pylori exposure by IgA and IgG ELISA tests. Clinical information, demographics and ELISA test results have been analyzed using SPSS.Version.18 (level of significance was less than 0.05).

**Results:**

Mean age of case group was 42.27±13.64 years; in control group it was 45.52±13.83 years. There was a significant difference between the case and control groups in IgG study of the following subgroups: age under 30, females, males, urban, higher education level and BMI between 18.5 and 24.9 (p-value was respectively; 0.004, 0.014, 0.047, 0.002, 0.013, 0.003). On the basis of logistic regression; IBD was less common in females, patients with lower education and patients with positive result of IgG (p-value was respectively 0.002, 0.013, 0.010).

**Conclusion:**

As a result of this study, Helicobacter pylori exposure, may could play a protective role against inflammatory bowel disease.

Glassified as Crohn’s disease and ulcerative colitis, IBDs have a recurrent-quiescent nature. They can cause tissue damage, surgical interventions, and failure or functional disabilities of the digestive system over the course of time. It is assumed that IBDs result from a complicated and unknown confrontation between environmental factors (such as infections, drugs, tobacco, food particles) and genetic factors of a host (1-4). Several studies have been conducted on its role in other conditions for example colon cancer. Helicobacter pylori plays a protective role against some autoimmune diseases such as asthma and diabetes mellitus type 1. The mechanism of this protective role is still unknown. However, it appears that a distinct expression of an acute or chronic immune response to topical mucosal inflammation affects it. This response releases cytokines systematically. On the contrary, it can regulate the systematic immune response and suppress self-immunity by banishing the response from Th1/Th17 (1, 2, 5). Derangement of host immune response to common bacteria is known as an important pathogenic mechanism in IBD. 

An increase has been observed in intestinal bacteria connected to intestinal epithelium in IBDs. This theory increases the probability that H. pylori can play a role in IBDs; On the other hand, H. pylori is seen more in communities with lower socioeconomic status. It becomes less prevalent by improving hygiene. However, IBD is observed more common in population with higher socioeconomic levels (6). Therefore, it appears that there must be an inverse relationship between the prevalence of helicobacter pylori and IBD (1, 2, 7-9). Moreover, a continuous increase has been reported in the emergence of ulcerative colitis in areas with endemic H. pylori and where helicobacter has been eradicated (2). 

In 2017, 41 studies were assessed and results showed the incidence and prevalence of Crohn’s disease that was inversely and significantly associated with prevalence of H. pylori infection. The studies were conducted in Europe, Japan, USA and Australia (10). The presence of specific antibodies of IgA and IgG classes in the serum against bacteria is a diagnostic factor of bacterial exposure and ELISA is a selective technique for diagnosing these antibodies (11). The aim of this study was to investigate the coincidence of H. pylori exposure in patients with active destructive colitis and in control group visiting endoscopy wards of teaching hospitals in Babol, Iran in 2015-2016. The socioeconomic factors were also included to assess association dependency to these factors due to insufficiency of data in this context.

## Methods

This case-control study was conducted on patients with the symptoms of IBDs (like abdominal pain, rectal bleeding and weight loss) visiting the endoscopy wards of teaching hospitals in Babol, Iran from March 20, 2015 to March 20, 2016 using the simple random sampling method. The patients were asked for written consent. Ethical approval for this study was obtained from the Ethics Committee of Babol University of Medical Sciences (MUBABOL.REC.1394.214). with presumption of 50% H. pylori seropositivity, there were 180 samples selected in total to identify the effect of 20% in H. pylori seropositivity at a 95% confidence level and 80% statistical power between the cases and control groups. Sixty cases were selected with new diagnosis of IBD (ulcerative colitis or Crohn’s disease) according to the evidence of IBD in both colonoscopy and pathology report of biopsy. A hundred twenty patients, whose colonoscopy and biopsy results not confirmed IBDs were selected as controls. 

Both groups were interviewed and if they had previous records of IBDs, former treatments for eradication of H. pylori, history of taking proton pump inhibitors, receiving sulfasalazine or type-2 histamine receptor blockers, antiretroviral drugs, taking any drugs affecting intestines, and any diagnosed concurrent intestinal infections, were excluded from the study.

The study data included demographics (age, gender, place of residence, weight, height, and educational attainment) and clinical information (records of taking proton pump inhibitors and sulfasalazine, eradication of helicobacter pylori, receiving type-2 histamine receptor blockers, antiretroviral drugs, and previous IBDs). To check the presence of helicobacter pylori exposure, 1 cc of venous blood was taken from all patients. Then an ELISA kit (made by Padtan Elm Co. with cutoff range of <20AU/ml negative, 20-30AU/ml equivocal and >30AU/ml positive) was used to measure the titrations of serum IgA and IgG antibodies against H. pylori through indirect ELISA. The results were recorded on the checklist. 

**Statistical analysis: **We used SPSS software Version 18. The chi-square and t-test were applied in bivariate analysis for categorical and continuous data, respectively. Multiple logistic regression model performed to estimate adjusted odds ratio of H. pylori seropositivity and its 95% confidence interval by controlling sex and educational level. In variable selection, we used stepwise method. In logistic regression model, the independent variables were helicobacter seropositivity versus negative, female versus male and education level (low versus high) that were defined as indicator variables. The dependent variable was case status (case versus control). The significance level was considered less than 0.05 for all cases.

## Results

The demographic findings are stated in table.1. Thirty one (51.7%) cases and 90 (75%) controls had positive IgG for H.pylori (more than 30 AU/ml) (P=0.002). 28 (46.7%) cases and 62 (51.7%) controls had positive IgA for H.pylori (more than 30 AU/ml) (P=0.635) (table 1).

There was a significant difference between cases and controls in H. pylori exposure based on IgG test in these subgroups: age below 30 years old (P=0.004), females (men with P=0.047 and women with P=0.014), urban residency (P=0.002), academic education (P=0.013), BMI between 18.5 and 24.9 (P=0.003). The female gender, lowest level of education (undergraduate) and positive results of IgG test for H.pylori were regarded as independent factors having protective roles in IBDs after moderating the intervener variables (P=0.002, 0.013, and 0.010) (table 2)

**Table 1:** Demographic characteristics and serologic findings of patients with IBD and the control group

**Variable**		**Group**		**P-value**
		**Case**	**Control**	
Gender	Male	41 (68.3%)	60 (50.0%)	0.007
	Female	19 (31.7%)	60 (50.0%)	
Age (year)	≤30	13 (21.7%)	12 (10.0%)	0.104
	30-50	32 (21.7%)	73(60.8%)	
	>50	15 (25.0%)	35 (29.2%)	
Place of Residence	Urban	32 (53.3%)	53 (44.2%)	0.270
	Rural	28 (46.7%)	67 (55.8%)	
Educational Attainment*	Illiterate	5 (8.3%)	19 (18.4%)	0.026
	Primary	12 (20.0%)	7 (6.8%)	
	High school	13 (21.7%)	30 (29.1%)	
	Academic education	30 (50.0%)	47 (45.6%)	
BMI Kg/m2	<18.5	10 (16.7%)	9 (7.5%)	0.165
	18.24-25.9	32 (53.3%)	61 (50.8%)	
	25.9-29	16 (26.7%)	47 (39.2%)	
	≥30	2 (3.3%)	3 (2.5%)	
IgG**	Positive	31 (51.7%)	90 (75.0%)	0.002
	Negative	29 (48.3%)	30 (25.0%)	
IgA**	Positive	28 (46.7%)	62 (51.7%)	0.635
	Negative	32 (53.3%)	58 (48.3%)	

* there were missing data from this item in control group.

** <20AU/ml negative 20-30AU/ml Equivocal >30AU/ml positive

**Table 2:** The adjusted odds ratio and its 95% confidence interval of associated factors with IBD risk using multiple logistic regression model with stepwise method

**Variable**	**Odds ratio**	95% CI*	**P-value**
Female versus male	0.33	0.16 ; 0.66	0.002
Low versus high graduation level	6.24	1.48 ; 26.24	0.013
Positive IgG versus negative IgG	0.39	0.19 ; 0.80	0.010

CI: confidence interval

## Discussion

The research findings indicate that exposure of H pylori has a protective role against IBDs. Many organisms are proposed as triggering factor in the pathogenesis of IBD but none of them have been proven. Gastrointestinal infections can make quick changes in the population of bacteria causing IBDs (2, 12, 13). In this regard, the role of H. pylori in ulcerative colitis has been investigated in many studies (2,14). El-Omar *et al.* reported the inverse relationship between H. pylori and IBD in 1994 for the first time. Then other studies attributed this relationship to the consumption of sulfasalazine. Nevertheless, different rates of prevalence were apparently independent of sulfasalazine. Another study indicated that the onset ages of IBD had nearly 10 years difference in patients with negative and positive H. pylori (15, 16). Ram *et al.* reported the relationship between H. pylori and IBDs (17). Nonetheless, Oliviera *et al. *and Parlak *et al*. did not find any relationship between H. pylori and ulcerative colitis (18, 19). In this study, the ELISA method for detection of anti-helicobacter IgG and IgA was used to identify H. pylori exposure. IgA showed no significant difference between cases and controls. However, IgG detection showed 75% of controls and 51.7% of cases had prior exposure to H. pylori. This statistically significant difference indicates a lower rate of helicobacter pylori exposure in patients with IBD compared to the control group. According to Jin *et al.*, the conditions of patients with UC got severed when H. pylori declined. This finding shows the negative relationship between H. pylori and ulcerative colitis. The possible hypothesis is the protective role of H. pylori against ulcerative colitis. Fewer UC patients were significantly reported to be affected by helicobacter pylori in comparison with the control group (2). Moreover, Jin *et al.* identified H. pylori infection through the urea breath test method a more acceptable method. 

Sladek *et al.* investigated 94 children who were newly diagnosed with IBD. The cases and controls were the same age and gender and in the same socioeconomic conditions. The results indicated that the rate of helicobacter pylori gastric colonization in child with IBD was significantly lower than the control group (9.6% compared with 38.4%). Helicobacter pylori gastric colonization was significantly higher in Crohn’s disease than in ulcerative colitis. There was no significant difference in the IBD average age of onset between positive and negative H. pylori groups. Finally, they stated that the prevalence of H. pylori was lower in IBD patients than in the control group, something which is not related to the eradication of helicobacter pylori because all the patients included in the study were newly-diagnosed, so they did not receive any treatments (20).

Song *et al.* reported a significant difference between patients with IBD and the control group in H. pylori (25.3% in cases and 52.5% in controls). Furthermore, there was a significant difference between ulcerative colitis (32%) and Crohn’s disease (17.7%). In IBD patients younger than 60 years old with the record of receiving metronidazole and ciprofloxacin, infection decreased significantly (13% and 6.7%, respectively). Although, those who did not receive any antibiotics were affected by H. pylori less often than the control group (22% in Crohn’s disease, 33.8% in ulcerative colitis, and 52.5% in the control group). Apart from the age, other demographic variables did not show any effects on results. Finally, they stated that the prevalence of H. pylori in patients with IBD, especially Crohn’s disease, was lower than in the control group. This finding indicates that H. pylori infection supposed to decrease the possible risks of IBD in younger adults (21).

Zhang *et al.* used PCR to investigate helicobacter pylori colonization in gastric tissue biopsies taken from patients with IBD. The results were not significantly different between IBD (10%) patients and the control (6.3%) group. In a cohort study conducted on 208 patients with IBD and 416 controls, the urea breath test was carried out with carbon 13. Significant results obtained from patients (19.7%) and control group (48.8%) in terms of H. pylori infection (22).

According to the findings of this study, IBD was significantly related to gender and educational attainment. However, it was not significantly related to the effects of BMI, place of residence, and age. Though, Jin *et al.* reported a lower rate of BMI in IBD patients.

Sonnenberg *et al.* conducted a pathology on 5493 individuals (550 patients with IBD and 4943 controls without IBD) during endoscopy-colonoscopy procedures. They stated that Helicobacter-negative gastritis and duodenitis is more prevalent in patients with IBD than healthy controls (23). Roka *et al.* conducted a study in 2014 and reported the prevalence of H. pylori in patients with IBD was significantly lower than the control group (3.8% to 13.2%). IBD patients were significantly older than those patients without IBD. According to their study, the prevalence of helicobacter pylori in children with IBD was lower than in the control group. This study confirmed an inverse relationship between H. pylori and IBD (24). Vare *et al.* reported that the prevalence of H. pylori was lower in IBD patients than in the control group (24% to 37%). The onset age of IBD was lower in seronegative patients. Finally, they stated that the prevalence of H. pylori was lower in IBD patients particularly in Crohn’s disease. This finding shows the protective role of H. pylori (25). In Denmark, Bartels *et al.* studied H. pylori in patients with Crohn’s disease, ulcerative colitis, and celiac through the urea breath test in a 6-year interval. They reported the low prevalence of Crohn’s disease in positive H. pylori cases compared with negative cases. They concluded that helicobacter pylori could play a protective role against Crohn’s disease (26). The possible explanations of these findings were proposed by many studies. A study suggested that H. pylori may prevent IBD by producing antibacterial peptide (27). Another study linked this role to IL-10 production in the mesenteric lymph nodes of the infected animals and to H. pylori DNA, which contains a high ratio of immuneregulatory to immunostimulatory sequences. (28). 

In the present study, the results of the logistic regression analysis showed independent protective role of female gender, lower educational state and positive IgG for H.pylori against IBD. These findings are probably due to other aspects of their lifestyle which affects IBD occurrence. As it has been mentioned in recently published article “it could mean that H. pylori is merely an indicator of a lifestyle protective against Crohn’s disease rather than a causal factor” (9). 

The main limitation of the present study was the small sample size because it was focused on cases who were newly diagnosed with IBDs. To conquer the problem and achieve more power to defeat the effect size of interest, we increased the sample size of control in turn. Another limitation of this study was that the gold standard test for H. pylori diagnosis is tissue sampling and this problem can be solved with dual endoscopic study but this would cause additional burdens of an invasive procedure to the patients who are against medical ethics. The alternative method is urea breath test but unfortunately we did not access to this profit in our centers. According to the results, H. pylori exposure could play a protective role against IBDs. IBD is less frequently seen in females and in a population with lower level of education.
